# Onward Virus Transmission after Measles Secondary Vaccination Failure

**DOI:** 10.3201/eid3009.240150

**Published:** 2024-09

**Authors:** Isaac Tranter, Nicolas Smoll, Colleen L. Lau, Dusty-Lee Williams, Deborah Neucom, Donna Barnekow, Amalie Dyda

**Affiliations:** University of Queensland, Herston, Queensland, Australia (I. Trantner, N. Smoll, C.L. Lau, A. Dyda);; Sunshine Coast Hospital and Health Service, Maroochydore, Queensland, Australia (N. Smoll, D.-L. Williams, D. Neucom, D. Barnekow)

**Keywords:** measles, vaccination, vaccine-preventable diseases, secondary vaccination failure, viruses, vaccines, Australia

## Abstract

Measles in persons with secondary vaccination failure (SVF) may be less infectious than cases in unvaccinated persons. Our systematic review aimed to assess transmission risk for measles after SVF. We searched PubMed, Embase, and Web of Science databases from their inception dates. Inclusion criteria were articles describing persons who were exposed to measles-infected persons who had experienced SVF. Across the included 14 studies, >3,030 persons were exposed to measles virus from SVF cases, of whom 180 were susceptible, indicating secondary attack rates of 0%–6.25%. We identified 109 cases of SVF from the studies; 10.09% (n = 11) of case-patients transmitted the virus, resulting in 23 further cases and yielding an effective reproduction number of 0.063 (95% CI 0.0–0.5). These findings suggest a remarkably low attack rate for SVF measles cases, suggesting that, In outbreak situations, public health management of unvaccinated persons could be prioritized over persons with SVF.

Measles virus is one of the most infectious pathogenic agents and has a basic reproduction number (R­_0_) of 12–18, indicating that each infected person could infect 12–18 other susceptible persons (*1*). In 2022, measles caused an estimated 136,000 deaths globally and predominantly affected unvaccinated persons and undervaccinated children <5 years of age (*1*). Despite the number of deaths, measles vaccination has averted an estimated 57 million deaths in the 22 years since 2000 (*1*). Because of exclusive interhuman transmission, the existence of an effective and safe live attenuated vaccine, and the absence of healthy carriers, measles is inherently an eradicable disease. By 2023, a total of 82 countries had achieved measles elimination through high immunization coverage (*2*).

Despite the effectiveness of measles-containing vaccines, infection remains possible in immunized persons. This phenomenon has come to be known as vaccination failure. Two types of vaccination failure have been documented. Primary vaccination failure (PVF) results from a person’s failure to produce any humoral response to viral antigen (nonseroconversion) and is thought to occur in 5% of vaccinees (*3*). Secondary vaccination failure (SVF) seems to occur 6–26 years after the last vaccine dose and is a result of waning or incomplete immunity. SVF occurs in 2%–10% of vaccinated persons (*4*).

Measles infection after SVF, also known as modified measles, is generally milder (i.e., less cough, coryza, conjunctivitis, or fever), is associated with lower viral loads, and has lower risk for complicated disease (*5*). This form of measles is thought to occur because of insufficient but not absent immune response. Stated differently, immunity is sufficient to curtail symptoms and viral replication but insufficient to prevent infection. Muted symptoms in this scenario makes identification of measles cases on the basis of classical features unreliable.

In the postelimination setting, cases of measles after vaccination failure make up a higher proportion of total cases. This situation occurs when fewer unvaccinated persons exist to acquire the infection and the only remaining susceptible persons are those experiencing vaccination failure (*6*). In addition, in settings where measles does not commonly circulate, vaccinated persons are not exposed to wild virus and hence do not receive a natural booster (*7*). In the endemic setting, vaccinated persons make up 3%–8% of measles cases (*4*), in contrast to 14%–57% of cases in postelimination settings (*4*). This gap is likely the product of SVF because of waning immunity and the absence of natural immune boosters, rather than a primary vaccination failure, which would not be affected by the prevalence of circulating virus.

No universally agreed upon definition for measles SVF exists; however, several methods of classification have been suggested. The best methods remain the serum detection of IgG after vaccination but before infection and the avidity enzyme immunoassay. Measles IgG develops later in the course of infection (typically 7–10 days postinfection) and persists for long periods (generally for life) (*8*). IgG avidity index can determine recent (low avidity IgG, <40%) or past (high avidity, IgG >60%) exposure to the measles virus (*9*). Persons experiencing SVF are characterized by early production of IgG (before day 7 of infection) with a high avidity index (*10*). IgM may be produced in both novel and breakthrough measles infections. However, the absence of IgM in the presence of IgG within 7 days of infection is indicative of prior exposure and is another indicator of SVF (*9*).

Persons with SVF cases have lower measles viral loads in bodily fluids than do unvaccinated persons. Cycle threshold (Ct) values of real-time reverse transcription PCR are a semiquantitative measure of measles RNA loads (*11*). Higher Ct values equate to lower numbers of measles RNA copies in a sample and hence lower transmissibility (*11*).

It has been hypothesized that, because of reduced symptomatology and lower viral loads, SVF patients are less likely to transmit the measles virus (*11*–*14*). Our review aimed to determine the risk for transmission by persons with SVF for 2 key reasons: measles occurring post-SVF will increasingly make up a greater proportion of total cases in the postelimination setting, and an understanding of the different transmission dynamics in preelimination versus postelimination settings will enable more precise design and implementation of outbreak responses.

## Methods

This systematic review followed a study protocol registered with the International Prospective Register of Systematic Reviews before the date of first search. We followed the Preferred Reporting Items for Systematic review and Meta-analyses guidelines for reporting (*15*).

### Eligibility

This review included original, nonreview articles, published in English or French. The report must have described a person or cohort of persons who were exposed to a laboratory-confirmed measles-infected person who had experienced SVF. We defined confirmed measles through 3 methods: PCR detection of measles virus, detection of a >4-fold increase in measles IgG titer in the absence of recent vaccination with a measles-containing vaccine, or detection of measles IgM in the absence of recent vaccination with a measles-containing vaccine. We defined recent vaccination as administration of a measles-containing vaccine within the preceding 8 days–8 weeks. Given the unreliable nature of clinical signs and symptoms in measles SVF cases, we did not include signs and symptoms in the case definition for the purpose of this analysis.

We defined SVF as measles infection despite serologic immunity after documented or reported immunization with a measles-containing vaccine. Evidence of serologic immunity included documentation of a positive measles IgG result before exposure, high avidity measles IgG postinfection (>60%), concurrent positive IgG and negative IgM results within 7 days of infection, or early positive IgG alone within 7 days of infection. For inclusion we required that the report specify the number of exposed persons who then had a laboratory-confirmed case of measles within the next 21 days.

The original study protocol sought only to included articles that reported an attack rate post-SVF. However, to ensure transmission risk was fully reviewed, we completed an amendment to the study protocol to include any study that discussed onward transmission.

### Search Strategy and Study Selection

We searched PubMed, Embase, and Web of Science databases from their respective dates of inception through May 31, 2023, when the search was conducted (Appendix 1). We also searched citation lists of review articles and studies that met inclusion criteria for articles not already included. We also sought input from subject matter experts to minimize the chances of relevant studies being missed. We uploaded all articles found through these search processes to Covidence software (https://www.covidence.org), and 2 authors (I.T. and A.D.) screened titles and abstracts and then full texts. We resolved conflicts through collaborative discussion and referral to a third reviewer when required.

### Data Extraction and Quality Assessment

One author (I.T.) extracted data from studies meeting the inclusion criteria. The data extracted included study setting, location, SVF case definition, SVF case numbers, onward transmissions, exposure population sizes, and susceptible population size (i.e., persons who had not received >1 dose of measles-containing vaccine, had not received postexposure prophylaxis [PEP], were immunocompromised, or had unknown vaccination status). In addition, we recorded data on the administration of PEP with either measles-containing vaccine or measles immunoglobulin to all contacts involved in the outbreak rather than only those in contact with the SVF case-patient. We used PEP data to serve as a proxy for the strength of public health response taken. We recorded Ct value and IgG avidity data, where available, as the mean value of SVF cases in the dataset. A second author (A.D.) checked extracted data; we resolved discrepancies through discussion.

We used Joanna Briggs Institute methodological quality of case series studies critical appraisal tool to assess both quality and risk for bias of the studies included (Appendix 2 Table 1). We assessed publication bias by using a quasi-funnel plot with the point estimate of effective reproduction number (R_eff_) on the x-axis and total cases of SVF on the y-axis. We assessed the presence of bias on the basis of distribution of results.

### Data Analysis

#### R_eff_ Calculation

R_eff_ is the expected number of secondary cases produced by a typical infected person during their entire infectious period. R_eff_ is used in situations where the exposed population has a nonzero proportion of nonsusceptible persons (by natural immunity or vaccination) or public health measures are in place (e.g., movement restrictions and PEP requirements) (*16*). This situation is often observed in the SVF studies performed in high-income countries with high vaccination rates.

#### Directly Calculated R_eff_

We estimated R_eff_ after SVF by using the direct calculation approach. To ensure this estimate was robust, we demonstrated that this methodology is equivalent to traditional methods of estimating reproductive numbers (e.g., survivor function and ordinary differential equations) (Appendix 1). We calculated the direct R_eff_ by using simple division of the total number of secondary measles cases by the total number of primary SVF cases.

#### Estimated R_eff_

We calculated the direct R_eff_ from each study in which SVF cases and total transmissions were reported. We then obtained the estimated R_eff_ by using bootstrapped median and bootstrapped 95% CIs (2.5%–97.5%). We used that method because there is no known sampling distributions for the direct R_eff_ and the intervals were not expected to be symmetric.

#### Secondary Attack Rate

We calculated the secondary attack rate by using the number of new measles cases arising from exposure to an SVF case-patient as the numerator. We used the susceptible population exposed to this SVF case-patient as the denominator.

## Results

**Figure 1:**
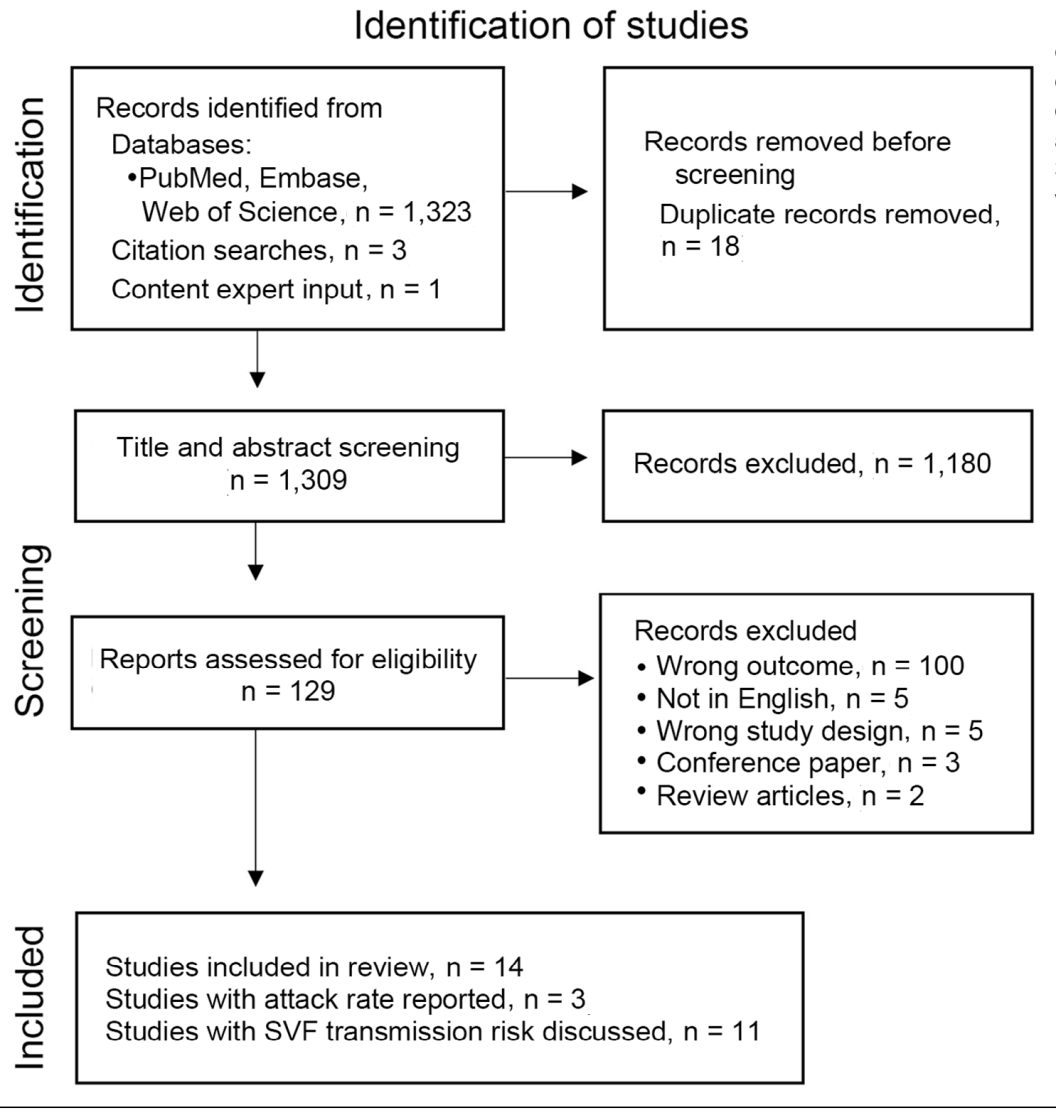
Flowchart of retrieved, excluded, and included items during systemic review of cases of onward virus transmission after measles SVF, as of March 31, 2023 (*15*). SVF, secondary vaccination failure.

The search yielded 1,327 articles, of which we removed 18 duplicates (Figure 1). We screened a total of 1,309 articles for inclusion, of which we excluded 1,295. We included a total of 14 studies in the final analysis. Three articles reported sufficient information from which to derive an attack rate (*17*–*19*). An additional 11 articles discussed transmission after measles SVF (Appendix 2 Table 2).

Nine studies were conducted in the healthcare setting, 1 in a military environment (*20*), and 4 in a community setting (*13*,*18*,*21*,*22*); 1 made reference to household contacts (*12*). All 14 studies were conducted in high-income or upper-middle–income countries.

Seven studies reported the administration of PEP with either measles-containing vaccine or measles immunoglobulin. Four of those studies reported administration of measles-containing vaccine (*12*,*13*,*20*,*23*), 3 studies reported measles immunoglobulin (*12*,*13*,*17*), and 2 studies reported administration of PEP but did not specify type (*18*,*19*).

Across the 14 studies, 109 cases of measles SVF had been identified (*4*,*12*,*13*,*17*–*27*). Of those cases, 11 (10.09%) were in persons who transmitted the virus, resulting in a total of 23 further measles cases (1–8 onward infections per transmitting case-patient) (*12*,*18*–*22*,*28*). Through the direct calculation method, those data yielded an R_eff_ of 0.211. The estimated R_eff_ was 0.063 (95% CI 0.0–0.5).

In the 6 studies that reported exposure population data, >3,030 persons were exposed to an SVF-affected person with measles during the infectious period (*17*–*20*,*23*,*27*); of those, 180 were considered susceptible (*17*–*19*). From the susceptible population, 5 infections occurred (*17*–*19*). We calculated a secondary attack rate from the 3 studies for which sufficient data were provided. The attack rate ranged from 0% (0/68) (*17*) to 6.25% (1/16) (*19*).

SVF was defined differently between studies. The most common method was high IgG avidity (77 persons) (*12*,*13*,*18*–*22*,*26*–*28*), followed by an IgM-negative or IgG-positive serologic profile (18 persons) (*24*,*25*). Thirteen persons had recorded measles IgG positivity before illness onset (*12*,*23*,*26*), and 1 person was classified on the basis of an early measles IgG result (day 2) (*17*).

Four studies reported Ct values from oropharyngeal samples, which ranged from 30.3 to 33.7 (*4*,*13*,*25*,*26*). Ten studies used IgG avidity to characterize SVF; 7 of those reported avidity values of 70.83%–88.6% (*12*,*13*,*18*–*20*,*22*,*26*).

**Figure 2:**
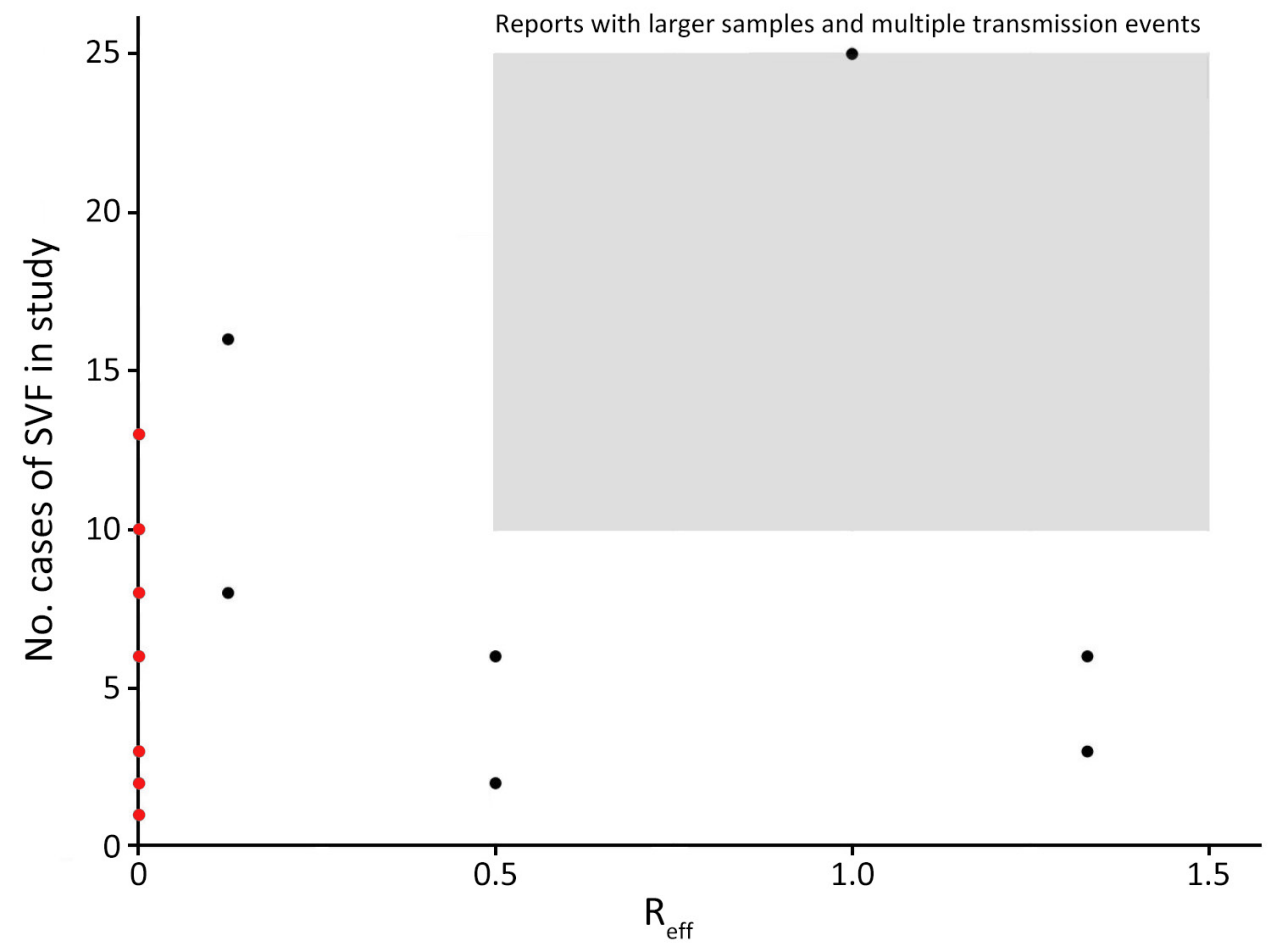
Study-specific effect size by total reported measles SVF cases identified during systemic review of cases of onward virus transmission after measles SVF, as of March 31, 2023. R_eff_, effective reproduction number; SVF, secondary vaccination failure.

We demonstrated the potential presence of publication bias as reviewed visually in the form of a quasi-funnel plot (Figure 2). Underreporting of outbreaks with large numbers of SVF cases with multiple transmission events may have occurred.

**Figure 3:**
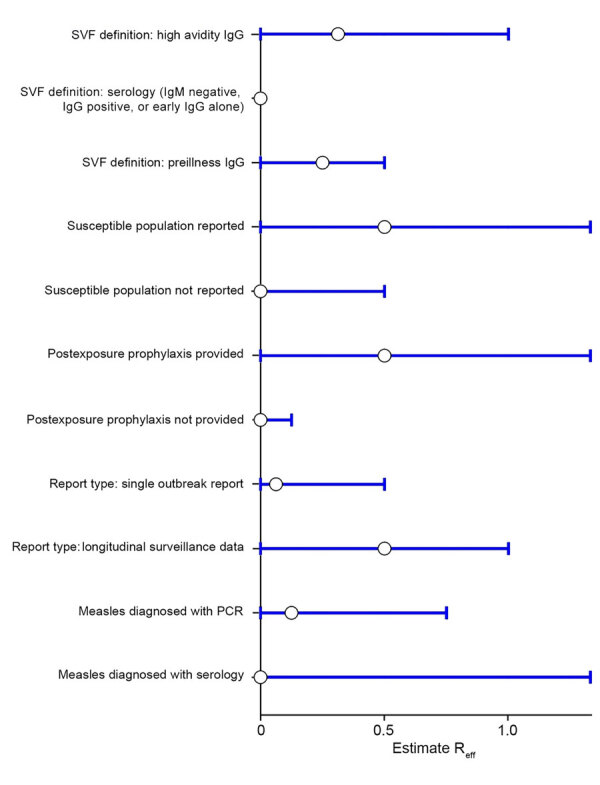
Sensitivity analysis of measles SVF cases identified during systemic review of cases of onward virus transmission after measles SVF, as of March 31, 2023. Error bars indicate 95% CIs. R_eff_, effective reproduction number; SVF, secondary vaccination failure; –ve, negative; +ve, positive.

Sensitivity analyses (Figure 3), in which the SVF case definition, measles case definition, and report type were varied, yielded similar results to the main analysis. Isolation of studies where PEP was provided to susceptible populations also yielded similar results to the main analysis.

## Discussion

This systematic review reports the attack rate and R_eff_ after measles SVF. We found 14 studies that reported the risk for measles infection after exposure to a person who experienced SVF. All included studies reported a very low attack rate (0%–6.25%) and R_eff_ (0.063 [95% CI 0.0–0.5]). Those findings suggests that persons with measles SVF have a very low risk for transmitting the disease.

Our findings are in keeping with the results of Gastañaduy et al. (*14*), who looked at the factors associated with measles transmission. Although they did not disaggregate PVF and SVF, they found an R_eff_ of 0.17 for persons who had received 1 dose of a measles-containing vaccine and an R_eff_ of 0.27 for those who received >2 doses of a measles-containing vaccines. That finding was in contrast to a R_eff_ of 0.76 for unvaccinated persons (*14*).

Although the overall attack rate for persons exposed to SVT case-patients appears to be low, prolonged exposure and confined settings probably result in higher risk for transmission. The presence of acutely unwell patients in healthcare settings appears to be the most likely scenario to result in infection (*12*,*13*,*17*–*19*,*22*,*23*,*26*,*27*). This probability stands to reason given the close and prolonged exposure that medical and nursing staff have with their patients, proximity to other patients, and the highly aerosolizing symptoms (i.e., cough) that brought those patients to seek medical care. Moreover, having household contacts, living in confined housing situations (e.g., military barracks, residential dormitories) (*12*,*20*,*29*), and being in educational settings (e.g., schools, universities) have also been documented as potentially high risk for transmission (*29*,*30*).

Ct values attained from our review compare appropriately with those found in other studies. Pacenti et al. (*31*) found vaccination failure (both PVF and SVF) Ct values of 27.6 (SD +4.8), whereas other authors reported median values of 32 (*13*). When comparing our result and those of other authors with unvaccinated controls (Ct 19.0–22.7) (*13*,*31*), we found that incidents of vaccine failure are more likely to have higher Ct values. These lower viral loads may be part of the explanation for the low attack rates attributable to SVF patients.

Another implication of this review is the observation that the attack rate after SVF appears to be exceptionally low. Although maintaining vigilance and appropriate measures remains crucial, the exceedingly low attack rate suggests that public health responses after SVF in high vaccination coverage regions could be implemented by using a transmission risk stratification approach. Because SVF case-patients are much less likely to transmit the virus, outbreak-control resources could be directed toward vaccine-naive and PVF-affected persons as a matter of priority. Public health follow-up will still be required for SVF-affected persons; however, such follow-up could occur once high transmission risk case-patients are managed. This approach would ensure the efficient use of resources, particularly during large outbreaks.

However, to properly inform any future public health responses, enhanced data collection and reporting surrounding the transmission dynamics after SVF is needed. Our study shows that data in this area are limited. To strengthen the evidence base, we advocate first for the development of a robust measles SVF case definition, then routine reporting of cases that meet that definition (*9*). In addition, reporting of exposed populations and attack rates is essential to create a more nuanced understanding of measles transmission from SVF case-patients. Standardizing data collection in this manner will render future research endeavors better equipped to analyze and interpret the implications of SVF, ultimately contributing to more effective and efficient public health strategies.

A key limitation of our study is potential publication bias. Underreporting of outbreaks that have high numbers of SVF but few or no reported transmission events is possible (Figure 2). This likelihood is consistent with investigators failing to report typical outbreaks (i.e., where persons with SVF transmit like persons without SVF). Another limitation of our analysis is the small sample size of studies available for analysis, which can make generalizability and confidence in the findings difficult and may not fully capture the variability and nuances of transmission after SVF. The lack of standardized criteria for defining SVF resulted in the exclusion of several potentially relevant studies from our analysis. The standard for defining SVF is the use of IgG avidity testing or preinfection serologic testing (*9*). Unfortunately, not all included studies had access to those diagnostic tools, instead relying on early infection course serologic test. Moreover, the reporting of exposed populations was deficient in many studies, limiting our ability to calculate an accurate attack rate. Of the studies that did report data on exposure populations, prior vaccination status was difficult to attain. In those instances, we classified persons who were unimmunized, were immunocompromised, or had any unknown vaccination status as susceptible. Thus, persons with unknown vaccinations status might have been vaccinated and therefore nonsusceptible, which could have led to an overestimation of this susceptible population size and an underestimation of the calculated attack rate. Furthermore, the studies included in our analysis were predominantly conducted in postelimination settings, which may limit the generalizability of our findings to measles-endemic settings. On the other hand, this fact may also be a strength, given that SVF is far more common in the postelimination setting, meaning those studies may more accurately reflect real-world scenarios.

Our findings suggest that the risk for onward transmission from persons with measles SVF is very low but not zero. In large outbreak situations, public health management of measles cases in unvaccinated persons could be prioritized before SVF cases. In postelimination settings, routine serologic testing for SVF, in addition to the standard PCR tests, may be a useful adjunct for risk stratification during outbreak management.

Appendix 1 Additional information about methodology used during system review of onward virus transmission after measles secondary vaccination failure.

Appendix 2 Additional information about case series appraisal and summary of measles secondary vaccination failure transmission risk studies.

## References

[1] World Health Organization. Measles. 2024 Jul 12 [cited 2024 Aug 1]. https://www.who.int/news-room/fact-sheets/detail/measles

[2] Patel MK, Goodson JL, Alexander JP Jr, Kretsinger K, Sodha SV, Steulet C, et al. Progress toward regional measles elimination—worldwide, 2000–2019. MMWR Morb Mortal Wkly Rep. 2020;69:1700–5. 10.15585/mmwr.mm6945a6 33180759 PMC7660667

[3] Anders JF, Jacobson RM, Poland GA, Jacobsen SJ, Wollan PC. Secondary failure rates of measles vaccines: a metaanalysis of published studies. Pediatr Infect Dis J. 1996;15:62–6. 10.1097/00006454-199601000-00014 8684879

[4] Fappani C, Gori M, Canuti M, Terraneo M, Colzani D, Tanzi E, et al. Breakthrough infections: a challenge towards measles elimination? Microorganisms. 2022;10:1567. 10.3390/microorganisms10081567 36013985 PMC9413104

[5] Hubiche T, Brazier C, Vabret A, Reynaud S, Roudiere L, Del Giudice P. Measles transmission in a fully vaccinated closed cohort: data from a nosocomial clustered cases in a teenage psychiatric unit. Pediatr Infect Dis J. 2019;38:e230–2. 10.1097/INF.0000000000002372 31261364

[6] Arima Y, Oishi K. Letter to the editor: measles cases among fully vaccinated persons. Euro Surveill. 2018;23:1800449. 10.2807/1560-7917.ES.2018.23.34.1800449 PMC6113742 30153884

[7] Markowitz LE, Preblud SR, Fine PE, Orenstein WA. Duration of live measles vaccine-induced immunity. Pediatr Infect Dis J. 1990;9:101–10. 10.1097/00006454-199002000-00008 2179836

[8] Gastañaduy PA, Clemmons N, Redd SB, Clemmons NK, Lee AD, Hickman CJ, et al. Measles [chapter 7]. In: Roush SW, Baldy LM, editors. Manual for the surveillance of vaccine-preventable diseases. Atlanta: Centers for Disease Control and Prevention; 2019 [cited 2024 May 16]. https://www.cdc.gov/vaccines/pubs/surv-manual/chpt07-measles.html

[9] Mercader S, Garcia P, Bellini WJ. Measles virus IgG avidity assay for use in classification of measles vaccine failure in measles elimination settings. Clin Vaccine Immunol. 2012;19:1810–7. 10.1128/CVI.00406-12 22971778 PMC3491540

[10] Javelle E, Colson P, Parola P, Raoult D. Measles, the need for a paradigm shift. Eur J Epidemiol. 2019;34:897–915. 10.1007/s10654-019-00569-4 31624970

[11] Seto J, Ikeda T, Tanaka S, Komabayashi K, Matoba Y, Suzuki Y, et al. Detection of modified measles and super-spreader using a real-time reverse transcription PCR in the largest measles outbreak, Yamagata, Japan, 2017 in its elimination era. Epidemiol Infect. 2018;146:1707–13. 10.1017/S095026881800211X 30081972 PMC9507958

[12] Kurata T, Kanbayashi D, Egawa K, Kinoshita M, Yoshida H, Miyazono M, et al. A measles outbreak from an index case with immunologically confirmed secondary vaccine failure. Vaccine. 2020;38:1467–75. 10.1016/j.vaccine.2019.11.075 31831219

[13] Sundell N, Dotevall L, Sansone M, Andersson M, Lindh M, Wahlberg T, et al. Measles outbreak in Gothenburg urban area, Sweden, 2017 to 2018: low viral load in breakthrough infections. Euro Surveill. 2019;24:1900114. 10.2807/1560-7917.ES.2019.24.17.1900114 31039835 PMC6628760

[14] Gastañaduy PA, Funk S, Lopman BA, Rota PA, Gambhir M, Grenfell B, et al. Factors associated with measles transmission in the United States during the postelimination era. JAMA Pediatr. 2020;174:56–62. 10.1001/jamapediatrics.2019.4357 31738391 PMC6865326

[15] Liberati A, Altman DG, Tetzlaff J, Mulrow C, Gøtzsche PC, Ioannidis JPA, et al. The PRISMA statement for reporting systematic reviews and meta-analyses of studies that evaluate health care interventions: explanation and elaboration. PLoS Med. 2009;6:e1000100. 10.1371/journal.pmed.1000100 19621070 PMC2707010

[16] Gostic KM, McGough L, Baskerville EB, Abbott S, Joshi K, Tedijanto C, et al. Practical considerations for measuring the effective reproductive number, R_t_. PLOS Comput Biol. 2020;16:e1008409. 10.1371/journal.pcbi.1008409 33301457 PMC7728287

[17] Jones J, Klein R, Popescu S, Rose K, Kretschmer M, Carrigan A, et al. Lack of measles transmission to susceptible contacts from a health care worker with probable secondary vaccine failure—Maricopa County, Arizona, 2015. MMWR Morb Mortal Wkly Rep. 2015;64:832–3. 10.15585/mmwr.mm6430a5 26247437 PMC5779579

[18] Rosen JB, Rota JS, Hickman CJ, Sowers SB, Mercader S, Rota PA, et al. Outbreak of measles among persons with prior evidence of immunity, New York City, 2011. Clin Infect Dis. 2014;58:1205–10. 10.1093/cid/ciu105 24585562

[19] Zhang Z, Chen M, Ma R, Pan J, Suo L, Lu L. Outbreak of measles among persons with secondary vaccine failure, China, 2018. Hum Vaccin Immunother. 2020;16:358–62. 10.1080/21645515.2019.1653742 31487215 PMC7062416

[20] Avramovich E, Indenbaum V, Haber M, Amitai Z, Tsifanski E, Farjun S, et al. Measles outbreak in a highly vaccinated population—Israel, July–August 2017. MMWR Morb Mortal Wkly Rep. 2018;67:1186–8. 10.15585/mmwr.mm6742a4 30359348 PMC6290812

[21] Iwamoto M, Hickman CJ, Colley H, Arciuolo RJ, Mahle CE, Deocharan B, et al. Measles infection in persons with secondary vaccine failure, New York City, 2018-19. Vaccine. 2021;39:5346–50. 10.1016/j.vaccine.2021.07.078 34393016

[22] Santibanez S, Prosenc K, Lohr D, Pfaff G, Jordan Markocic O, Mankertz A. Measles virus spread initiated at international mass gatherings in Europe, 2011. Euro Surveill. 2014;19:6–15. 10.2807/1560-7917.ES2014.19.35.20891 25210982

[23] Gohil SK, Okubo S, Klish S, Dickey L, Huang SS, Zahn M. Healthcare workers and post-elimination era measles: lessons on acquisition and exposure prevention. Clin Infect Dis. 2016;62:166–72. 10.1093/cid/civ802 26354971 PMC4723666

[24] Edmonson MB, Addiss DG, McPherson JT, Berg JL, Circo SR, Davis JP. Mild measles and secondary vaccine failure during a sustained outbreak in a highly vaccinated population. JAMA. 1990;263:2467–71. 10.1001/jama.1990.03440180073035 2278542

[25] Gibney KB, Attwood LO, Nicholson S, Tran T, Druce J, Healy J, et al. Emergence of attenuated measles illness among IgG-positive/IgM-negative measles cases: Victoria, Australia, 2008–2017. Clin Infect Dis. 2020;70:1060–7. 10.1093/cid/ciz363 31056637

[26] Hahné SJM, Nic Lochlainn LM, van Burgel ND, Kerkhof J, Sane J, Yap KB, et al. Measles outbreak among previously immunized healthcare workers, the Netherlands, 2014. J Infect Dis. 2016;214:1980–6. 10.1093/infdis/jiw480 27923955

[27] Rota JS, Hickman CJ, Sowers SB, Rota PA, Mercader S, Bellini WJ. Two case studies of modified measles in vaccinated physicians exposed to primary measles cases: high risk of infection but low risk of transmission. J Infect Dis. 2011;204(Suppl 1):S559–63. 10.1093/infdis/jir098 21666213

[28] Bianchi S, Gori M, Fappani C, Ciceri G, Canuti M, Colzani D, et al. Characterization of vaccine breakthrough cases during measles outbreaks in Milan and surrounding areas, Italy, 2017–2021. Viruses. 2022;14:141068. 10.3390/v14051068 35632809 PMC9147195

[29] Paunio M, Peltola H, Valle M, Davidkin I, Virtanen M, Heinonen OP. Explosive school-based measles outbreak: intense exposure may have resulted in high risk, even among revaccinees. Am J Epidemiol. 1998;148:1103–10. 10.1093/oxfordjournals.aje.a009588 9850133

[30] Smith J, Banu S, Young M, Francis D, Langfeldt K, Jarvinen K. Public health response to a measles outbreak on a university campus in Australia, 2015. Epidemiol Infect. 2018;146:314–8. 10.1017/S0950268817003089 29338804 PMC9134550

[31] Pacenti M, Maione N, Lavezzo E, Franchin E, Dal Bello F, Gottardello L, et al. Measles virus infection and immunity in a suboptimal vaccination coverage setting. Vaccines (Basel). 2019;7:199. 10.3390/vaccines7040199 31795157 PMC6963570

