# Supplementary material for: Onward Virus Transmission after Measles Secondary Vaccination Failure
Source: Emerg Infect Dis. 2024 Sep;30(9):1747–54. doi: 10.3201/eid3009.240150 (PMC11346981; doi:10.3201/eid3009.240150)
Supplement: Appendix 1 — Additional information about methodology used during system review of onward virus transmission after measles secondary vaccination failure. [file 24-0150-Techapp-s1.pdf]

Article DOI: https://doi.org/10.3201/eid3009.240150

EID cannot ensure accessibility for supplementary materials supplied by authors. Readers who have difficulty accessing supplementary content should contact the authors for assistance.

Onward Virus Transmission after Measles Secondary Vaccination Failure

Appendix 1

PubMed

(“previous immunity”[tiab] OR “prior immunity”[tiab] OR “vaccination failure”[tiab] OR “vaccine failure”[tiab] OR “breakthrough infection*”[tiab] OR immunized[tiab] OR immunised[tiab]) AND (Measles[Mesh] OR Measles[tiab] OR “Measles Vaccine”[Mesh] OR “Measles-Mumps-Rubella Vaccine”[Mesh])

Web of Science

(“previous immunity” OR “prior immunity” OR “vaccination failure” OR “vaccine failure” OR “breakthrough infection*” OR immunized OR immunised) AND (Measles OR Measles OR “Measles Vaccine” OR “Measles-Mumps-Rubella Vaccine”)

Embase

('previous immunity':ti,ab OR 'prior immunity':ti,ab OR 'vaccination failure':ti,ab OR 'vaccine failure':ti,ab OR 'breakthrough infection*':ti,ab OR immunized:ti,ab OR immunised:ti,ab) AND (Measles/exp OR Measles:ti,ab OR 'Measles Vaccine'/exp OR 'Measles-Mumps-Rubella Vaccine'/exp)

Relationship of Directly Calculated R_{eff} to Survivor function R_{eff} and Ordinary Differential Equations R_{eff}.

R_{eff} can also be estimated using the survivor function (*1*–*3*) and ordinary differential equations (ODEs). In both the survivor function (equation with integral) and the ODE (equation

with βIS) the $b(x)$ can be simplified to 1, meaning 100% chance the person will transmit throughout their infective period. For these models there can be 1 or more infected individuals transmitting (I).

Survivor Function and Ordinary Differential Equations

$$R_{eff} = I\int_0^a f(x)b(x)dx = \beta IS \cdot b(x) \cdot a$$

$f(x)$ = transmissions function to susceptible individuals per unit of time for period a (during one infective period, which is ≈8 days for measles; see Figure 3). $f(x)$ is similar to β in that it represents a rate, but the rate of $f(x)$ changes overtime. Integration (area under the curve) represents the total transmissions during the period a.

$b(x)$ = probability the person is infective during entire infective period a (8 days). $b(x)$ is a term that can be a simple proportion, or a time dependant function. This term modifies the likelihood of being infectious, similar to how an individual with SVF will have a modified transmission period. The function $b(x)$ may modify the transmission rate profile or make it an all or none phenomenon. $b(x)$ is a unique function, which is most thought of as 1 in ODEs (thus not included), as all infected persons always transmit in the common models. However, not all persons with SVF transmit the virus, which is why the term $b(x)$ should be included or modeled when estimating the R_{eff} for persons with SVF.

a = duration of infective period. Measles is generally infective from 4 days before the onset of the rash, and 4 days after the onset of the rash, giving an infective period of 8 days (a = 8 days).

βIS = the transmission rate per unit of time (β), which in this case will be 1 day. I is the proportion of persons that are infective. The proportion of persons that are susceptible is represented by S and is equal to $1 - I$. The following must hold true: $S + I = 1$, and if there are 100 persons in the model, then $S = 0.99$ and $I = 0.01$. The total transmissions per day is then multiplied by the infectious period a to provide an estimate of total transmissions per infective period R_{eff} β the transmission rate, assumed to be fixed for the entire infective period in ordinary differential equations (ODE) here.

References

1. Heesterbeek JAP, Dietz K. The concept of R_0 in epidemic theory. Stat Neerl. 1996;50:89–110. https://doi.org/10.1111/j.1467-9574.1996.tb01482.x

2. Heffernan JM, Smith RJ, Wahl LM. Perspectives on the basic reproductive ratio. J R Soc Interface. 2005;2:281–93. PubMed https://doi.org/10.1098/rsif.2005.0042

3. Li J, Blakeley D, Smith RJ. The failure of R_0. Comput Math Methods Med. 2011;2011:527610. PubMed https://doi.org/10.1155/2011/527610

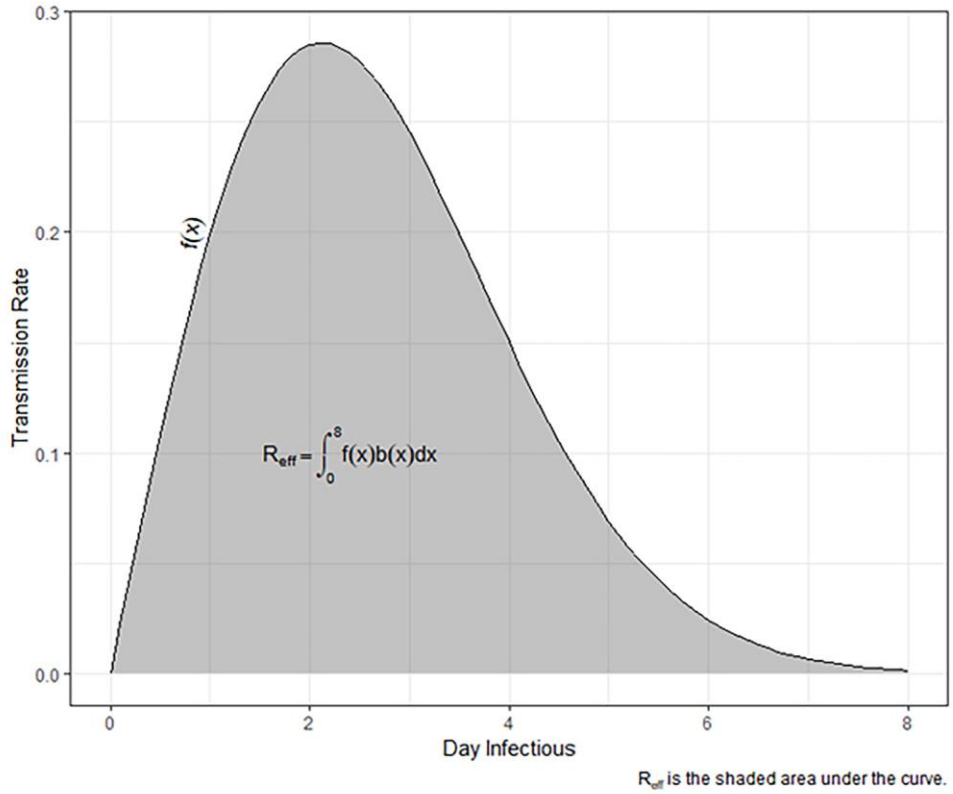

Appendix 1 Figure. Example curve of the function of the transmission rate during the infectious period. This curve represents the probability of transmission on a given day and assumes an average number of contacts per day, whereas the ODE parametrizes this separately. The transmission rate function $f(x)$ bears similarity to β used in ODEs, except that β would remain uniform throughout the infectious period and it assumes a fixed number of contacts per day (IS component of the βIS). In this example, the person is most infectious at the end of day 1 to about day 3. This curve would look similar (but not identical) to a curve representing excreted live viral load. The R_{eff} in its most basic form for one person that is infectious can be taken as the integral of the function shown in Figure 3. In other words, the R_{eff} is the area under the curve.
